# Antibiotic-Induced Mutagenesis: Under the Microscope

**DOI:** 10.3389/fmicb.2020.585175

**Published:** 2020-10-22

**Authors:** Sarah A. Revitt-Mills, Andrew Robinson

**Affiliations:** ^1^Molecular Horizons Institute and School of Chemistry and Molecular Bioscience, University of Wollongong, Wollongong, NSW, Australia; ^2^Illawarra Health and Medical Research Institute, Wollongong, NSW, Australia

**Keywords:** stress response, DNA damage, imaging, experimental evolution, recombination

## Abstract

The development of antibiotic resistance poses an increasing threat to global health. Understanding how resistance develops in bacteria is critical for the advancement of new strategies to combat antibiotic resistance. In the 1980s, it was discovered that certain antibiotics induce elevated rates of mutation in bacteria. From this, an “increased evolvability” hypothesis was proposed: antibiotic-induced mutagenesis increases the genetic diversity of bacterial populations, thereby increasing the rate at which bacteria develop antibiotic resistance. However, antibiotic-induced mutagenesis is one of multiple competing factors that act on bacterial populations exposed to antibiotics. Its relative importance in shaping evolutionary outcomes, including the development of antibiotic resistance, is likely to depend strongly on the conditions. Presently, there is no quantitative model that describes the relative contribution of antibiotic-induced mutagenesis to bacterial evolution. A far more complete understanding could be reached if we had access to technology that enabled us to study antibiotic-induced mutagenesis at the molecular-, cellular-, and population-levels simultaneously. Direct observations would, in principle, allow us to directly link molecular-level events with outcomes in individual cells and cell populations. In this review, we highlight microscopy studies which have allowed various aspects of antibiotic-induced mutagenesis to be directly visualized in individual cells for the first time. These studies have revealed new links between error-prone DNA polymerases and recombinational DNA repair, evidence of spatial regulation occurring during the SOS response, and enabled real-time readouts of mismatch and mutation rates. Further, we summarize the recent discovery of stochastic population fluctuations in cultures exposed to sub-inhibitory concentrations of bactericidal antibiotics and discuss the implications of this finding for the study of antibiotic-induced mutagenesis. The studies featured here demonstrate the potential of microscopy to provide direct observation of phenomena relevant to evolution under antibiotic-induced mutagenesis.

## Antibiotic-Induced Mutagenesis: From Molecules to Cells to Populations

Mutation is one of the two major mechanisms for the development of antibiotic resistance in bacteria (Woodford and Ellington, 2007). In the laboratory, high-level resistance against most antibiotics can be acquired by bacteria through the development of mutations (Woodford and Ellington, 2007). In clinically relevant bacterial pathogens, resistance to most antibiotics is acquired through lateral gene transfer; however, mutational resistance dominates in some circumstances. Resistance against fluoroquinolones (Appelbaum and Hunter, 2000; Ruiz, 2003), rifampicin (Gillespie, 2002), oxazolidinones (Long and Vester, 2012), fusidic acid (Nagaev et al., 2001; Besier et al., 2005), and streptomycin (Gillespie, 2002) frequently arises through mutation (Woodford and Ellington, 2007). Commonly these are “target-gene mutations,” in which the systems that are targeted by the antibiotic become altered in such a way that the antibiotic no longer binds to its target or is otherwise made ineffective. Another important class of mutations that occur commonly among clinical isolates is loss-of-function mutations in regulators that can lead to increased activity of associated multi-drug efflux pumps (Woodford and Ellington, 2007). Interestingly, the causative agent of tuberculosis, *Mycobacterium tuberculosis*, appears to acquire antibiotic resistance exclusively through mutation, as opposed to lateral gene transfer (Gillespie, 2002).

In many cases, we have a strong understanding of how particular mutations lead to an increase in resistance against a particular antibiotic (Woodford and Ellington, 2007). Much is known about the molecular mechanisms that generate mutations and the evolutionary dynamics that allow them to fix within bacterial populations (Andersson and Hughes, 2017). In the simplest scenario, mutations occur spontaneously, arising from errors made during DNA synthesis (Martinez and Baquero, 2000). Mutants are present in the population prior to antibiotic exposure and are strongly selected for once the population is exposed (Martinez and Baquero, 2000). This spontaneous mutation model serves as the null hypothesis in any study seeking to uncover the origins of a particular antibiotic resistance mutation. It is known, however, that some antibiotics can induce higher mutation rates in bacteria (Mao et al., 1997; Oliver et al., 2000; Blazquez et al., 2018). The role of induced mutagenesis in the evolution of antibiotic resistance remains a lively field of research (Blazquez et al., 2018).

Exposure to antibiotics can elevate mutation rates in bacteria in two ways. Both mechanisms have demonstrated capacity to drive the development of antibiotic resistance in laboratory models (Cirz et al., 2005; Pribis et al., 2019). First, antibiotic exposure often leads to the selection of hypermutator phenotypes; cells that have developed constitutively high mutation rates (Mao et al., 1997; Oliver et al., 2000; Shaver et al., 2002; Blazquez, 2003; Durao et al., 2018). These usually arise from loss-of-function mutations in systems that normally help to maintain low mutation rates, such as mismatch repair enzymes or proofreading exonucleases (Miller et al., 1999; Rodriguez-Rojas et al., 2013; Durao et al., 2018). Second, exposure to antibiotics can induce elevated mutation rates directly, by promoting the activities of error-prone DNA repair systems or by temporarily down-regulating mismatch repair (Frisch et al., 2010; Shee et al., 2011a). It is this second class of phenomena, termed antibiotic-induced mutagenesis or transient hypermutation, that is the primary focus of this review.

Antibiotic-induced mutagenesis is most often linked to the SOS response; a transcriptional program that upregulates a set of genes (~40 in *Escherichia coli*), most of which have functions relevant to DNA damage (Blazquez et al., 2018; Maslowska et al., 2019). Included in the SOS regulon are genes that encode error-prone DNA polymerases (Goodman and Woodgate, 2013; Joseph and Badrinarayanan, 2020). During the SOS response, error-prone polymerases increase the rate at which mutations appear in cells (Kuban et al., 2004; Hastings et al., 2010; Blazquez et al., 2018). In this way, antibiotic exposure begets mutagenesis. The observation of antibiotic-induced mutagenesis led to the proposal of an “increased genetic diversity” hypothesis in the early 2000s: antibiotic-induced mutagenesis drives an increase in the genetic diversity of bacterial populations and thus speeds the development of mutational antibiotic resistance (Radman, 1999; Martinez and Baquero, 2000; Rosenberg, 2001; Cirz et al., 2005). Twenty years later, this idea remains an active field of study (Blazquez et al., 2018).

There is ample evidence that antibiotic-induced mutagenesis occurs. Much of this evidence has been reviewed in detail recently (Blazquez et al., 2018). The primary question facing the field is: does it matter? Does the potential increase in mutation supply brought on by antibiotic-induced mutagenesis ever outweigh the loss of diversity brought on by the antibiotic killing the cells? To our knowledge, only one experimental evolution study has specifically investigated whether antibiotic-induced mutagenesis can play a dominant role in the evolution of antibiotic resistance. This study, which monitored the *de novo* appearance of ciprofloxacin-resistance mutations in a mouse infection model strongly suggested that resistance was dependent on antibiotic-induced mutagenesis (Cirz et al., 2005). As detailed below, assessing the role of antibiotic-induced mutagenesis in evolution requires that the effects of an antibiotic on mutagenesis are experimentally isolated from its effects on cell survival. Currently, this is almost impossible to do in animal models and this ultimately limits the use of top-down approaches toward studying evolution under antibiotic-induced mutagenesis. At the other end of the spectrum, observations made at the level of individual cells and small populations of cells may provide sufficient insight to enable accurate computer modeling of events that are too complex to monitor directly. With enough data, collected under carefully controlled conditions, this offers a potential means to approach the antibiotic-induced mutagenesis problem from the bottom-up.

**Figure 1:**
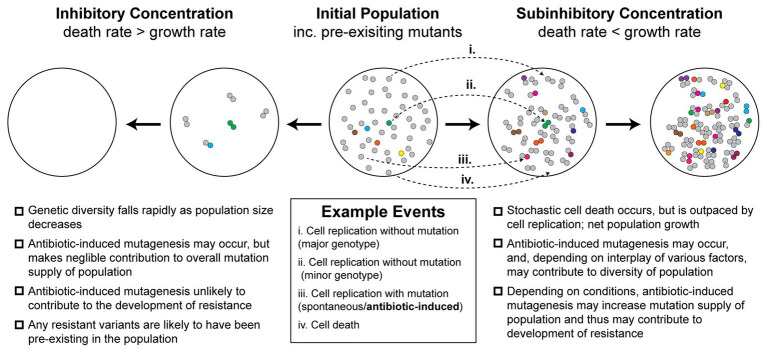
Antibiotic-induced mutagenesis is one of many factors that influence genetic diversity in bacterial populations. Many antibiotics induce elevated rates of mutagenesis in bacterial cells. However, the relative importance of antibiotic-induced mutagenesis in shaping the evolution of antibiotic resistance remains unclear. This figure illustrates some of the competing factors that increase and decrease genetic diversity (i.e., number of unique mutants) in antibiotic-exposed bacteria. It is not intended to serve as a functional model of resistance development. An initial population of antibiotic-naïve cells appears in the center. The majority of cells in the population are of the major genotype (“wild type”; gray circles). Due to there being a basal rate of spontaneous mutagenesis, some cells have variant genotypes (colored circles). The left panels depict a scenario in which a lethal dose of bactericidal antibiotics is administered. Under these conditions, the rate of cell death outpaces the rate of cell division. In the first stage of this inhibitory concentration scenario, most of the population is killed (circles disappear). A few cells initially survive long enough to divide, however, in time these are also killed. Thus, no cells remain alive by the second stage of the scenario. Because there is little cell growth, there is little scope for antibiotic-induced mutagenesis. The genetic diversity of the population crashes as rapid cell death dominates over other factors. Any antibiotic-resistant mutants that are selected under these conditions are likely to be pre-existing in the population prior to antibiotic exposure. In the second scenario, depicted in the two right panels, a sub-inhibitory concentration of a bactericidal antibiotic is applied. As demonstrated recently (Coates et al., 2018), many cells die under these conditions, however, the rate of cell division outpaces the rate of cell death, leading to net population growth. In the first stage of this sub-inhibitory concentration scenario, a portion of cells die (~30%), while the remaining cells divide (~70%). Antibiotic-induced mutagenesis increases the probability that variants are produced during these cell divisions (gray circles more often yield colored circles). Specific examples of each event are depicted with arrows (i–iv; see example events legend). These processes of stochastic cell death, cell division, and mutagenesis are repeated in the second stage. Over time, the population becomes larger and contains a greater number of variants than the initial population. It is important to note, however, that the number of cell divisions required to reach a particular population size will be higher than what would be required in the absence of drug. Many experimental measures of induced mutagenesis rates neglect this behavior and will therefore be overestimated if cell death is occurring frequently (Frenoy and Bonhoeffer, 2018). Nevertheless, antibiotic-induced mutagenesis can significantly contribute to the development of antibiotic resistance *in vivo* (Cirz et al., 2005). Overall, the genetic diversity of the population in the sub-inhibitory concentration scenario will depend on the complex interplay between cell death, cell division, spontaneous mutagenesis, and induced mutagenesis. Selection for antibiotic-resistant phenotypes will occur at all stages and is likely to involve additional complexity arising from population-level phenomena that are not depicted in the figure.

Antibiotic-induced mutagenesis will only influence evolutionary outcomes in situations where the bacterial cells remain alive long enough to produce new mutations. Thus, it is likely that it occurs infrequently under high antibiotic concentrations, where most cells die quickly. For this reason, antibiotic-induced mutagenesis is typically studied at antibiotic concentrations close to, but below, the minimum inhibitory concentration (MIC). The evolutionary dynamics at play within this near-MIC regime are more complex than those that occur at lethal concentrations of antibiotic (Figure 1). At concentrations near the MIC, selection for resistant variants will be weaker than for concentrations above MIC, although it is important to note that many antibiotics remain selective at concentrations far below the MIC (Andersson and Hughes, 2014). Near to the MIC, population genetics will play a major role in determining evolutionary outcomes (Hughes and Andersson, 2017). Competition for resources between variants (clonal interference) will play a large role in determining the population structure (Hughes and Andersson, 2017). Population size will also shape evolutionary outcomes – large populations tend to disfavor the selection of rare variants unless they are particularly advantageous (Hughes and Andersson, 2017). The relative rates of cell growth and cell death will also be important (Coates et al., 2018). It is widely known that exposing cells to near-MIC concentrations of bactericidal antibiotics causes the population to grow at a diminished rate. However, it was only demonstrated recently that this occurs because a portion of the population undergoes stochastic cell death (Coates et al., 2018). Thus, the population growth rate slows because the cell death rate approaches the cell growth rate, rather than all the cells simply growing at a slower rate. This phenomenon is depicted in Figure 1 and is expanded upon in a later section. At the same time that antibiotic-induced mutagenesis is acting to increase genetic diversity (i.e., number of unique mutants) within the population, cell death acts to reduce the size of the population. Evolutionary outcomes, including the likelihood of the population becoming antibiotic resistant, will depend strongly on the balance of these two parameters.

We propose that to better understand the role of antibiotic-induced mutagenesis on the evolution of antibiotic-resistance mutations, we need to be able to measure its effects while simultaneously monitoring cell and population level behaviors. This is not achievable with conventional microbiology techniques (e.g., fluctuation analyses), which operate as end-point assays and have indirect read-outs.

In recent years, researchers have begun to explore microscopy as a means to obtain direct, real-time observations of individual cells, and even individual molecules within them. The purpose of the current review is to highlight studies in which microscopy has improved our understanding of either the molecular mechanisms underlying antibiotic-induced mutagenesis or cell‐ and population-level events that occur when bacteria are challenged with sub-inhibitory concentrations of antibiotics. Much of the microscopy work presented here has been conducted in *E. coli*, due to its prevalence as a laboratory model organism, and with fluoroquinolones because these drugs are strongly mutagenic and are commonly utilized as model compounds in laboratory experiments. For a more comprehensive review of antibiotic-induced mutagenesis, which includes work in other systems, we suggest a recent review by Blazquez et al. (2018). There remains tremendous scope to extend the studies highlighted within this review to other organisms and other antibiotics. While microscopy cannot resolve all of the gaps in knowledge that remain open in the field of antibiotic-induced mutagenesis, the studies highlighted in the current review demonstrate the potential for enormous gains to be made. We anticipate that the greatest impact of microscopy will be in improving our understanding of how mutation rates are modulated in bacterial cells and in mapping mutations to fitness effects.

## The Cellular Context of Antibiotic-Induced Mutagenesis

**Figure 2:**
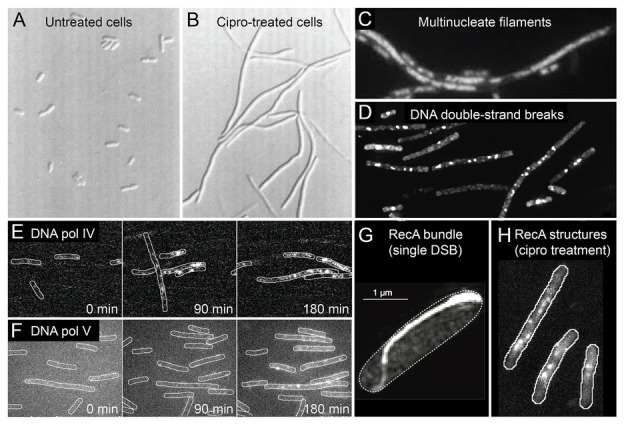
Conditions inside bacterial cells undergoing antibiotic-induced mutagenesis. With the exception of panels **(A)** and **(G)**, all panels depict *Escherichia coli* cells exposed to ciprofloxacin, a bactericidal antibiotic of the fluoroquinolones class. **(A,B)** *E. coli* growing as rod-shaped cells in the absence of antibiotics **(A)** and as filamentous cells in the presence of ciprofloxacin **(B)**. Figure adapted from Diver and Wise (1986). **(C)** Filamentous *E. coli* cells contain multiple copies of the chromosome, as shown by DAPI staining. The number of chromosomes scales with filament length. Figure adapted from Bos et al. (2015). **(D)** Large numbers of DNA double-strand breaks in ciprofloxacin-treated cells, as detected by a fluorescent protein fusion of the Gam protein. Figure adapted from Pribis et al. (2019). **(E,F)** Increased activity of fluorescently labeled error-prone DNA polymerases IV **(E)** and V **(F)** in ciprofloxacin-treated cells, as visualized by single-molecule fluorescence microscopy. Figures adapted from Henrikus et al. (2018, 2020). **(G)** Large bundle of fluorescently labeled RecA formed in an *E. coli* cell that contains a single DNA double-strand break induced *via* expression of the SceI restriction enzyme. Figure adapted from Lesterlin et al. (2014). **(H)** Colocalisation of error-prone DNA polymerase IV with RecA in ciprofloxacin-treated cells. RecA-ssDNA nucleoprotein filaments (RecA*) are specifically visualized using a portion of the RecA*-binding cI repressor protein that has been fused to a fluorescent protein. Figure adapted from Henrikus et al. (2020).

Before discussing the molecular mechanisms of antibiotic-induced mutagenesis, it is useful to consider the cellular context in which it takes place. Elevated mutagenesis is induced by several classical DNA-damaging agents, including UV light, mitomycin c, and methyl methanesulfonate (Maslowska et al., 2019). It is likely that all the antibiotics that induce mutagenesis do so by causing DNA damage. This can occur as a direct consequence of their mode of action (as is the case for fluoroquinolones, which inhibit type II-topoisomerases). Alternatively, DNA damage can occur indirectly, through errant metabolic processes that generate reactive-oxygen species (ROS; Kohanski et al., 2010). In Figure 2, we summarize some of the events known to take place in *E. coli* cells treated with ciprofloxacin, a fluoroquinolone antibiotic that is a key model compound in the study of antibiotic-induced mutagenesis (Cirz et al., 2005; Zhang et al., 2011; Bos et al., 2015; Pribis et al., 2019). It is likely that much of what we summarize for *E. coli* cells treated with ciprofloxacin extends to other organisms and to other antibiotics, although in most cases this remains to be tested.

Broadly speaking, *E. coli* cells respond to ciprofloxacin the same way they do to sub-inhibitory doses of other DNA damaging agents – they induce the SOS response (Pribis et al., 2019). SOS induction has been measured directly for several fluoroquinolones (Ysern et al., 1990; Thi et al., 2011; Pribis et al., 2019; Rodriguez-Rosado et al., 2019), as well as for the antifolates trimethoprim and sulfamethoxazole (Thi et al., 2011; Blazquez et al., 2012), and representative members of the β-lactams (Thi et al., 2011; Blazquez et al., 2012). Microscopy has revealed much about how *E. coli* cells respond to ciprofloxacin treatment. Once exposed, the cells continue to grow, but divide much less often, causing them to form long filaments (Figures 2A,B; Diver and Wise, 1986; Thi et al., 2011; Bos et al., 2015; Henrikus et al., 2018; Pribis et al., 2019). Bulk DNA replication continues during sub-inhibitory ciprofloxacin treatment (likely at a diminished rate; Goss et al., 1965; Snyder and Drlica, 1979) and as a result the filamentous cells contain multiple nucleoid masses (Figure 2C; Bos et al., 2015). This suggests that filamentous cells contain multiple independent (or semi-independent) copies of the bacterial chromosome, although it is important to note that the masses are less organized than the nucleoids of untreated cells, and the study did not examine whether masses represent intact or partially degraded nucleoids.

Cells treated with ciprofloxacin contain elevated numbers of DNA double-strand breaks, which can be detected using fluorescent fusions of the Gam protein from bacteriophage λ (Figure 2D; Shee et al., 2013; Pribis et al., 2019). Detection of double-strand breaks through a fluorescent protein fusion of the RecN protein has also been reported (Hong et al., 2017). It is likely that cells treated with other bactericidal compounds also contain large numbers of breaks as a consequence of having elevated ROS levels (Liu et al., 2010; Foti et al., 2012; Giroux et al., 2017). We have detected significant numbers of breaks appearing in cells treated with trimethoprim, for example (Henrikus et al., 2020). Double-strand breaks are important lesions for antibiotic-induced mutagenesis for multiple reasons. First, double-strand breaks are considered to be lethal to cells if left unrepaired (Cox, 2013). Second, the processing of double-strand breaks by the RecBCD helicase-nuclease complex is one of the major triggers for the induction of the SOS response in *E. coli* (Simmons et al., 2008). This probably extends to other organisms such as *Bacillus subtilis*, that uses the functional analog of RecBCD, AddAB (Simmons et al., 2008). Third, much of the increased mutagenesis associated with ciprofloxacin treatment in *E. coli* can be attributed to a form of error-prone double-strand break repair (Pribis et al., 2019). This activity requires error-prone DNA polymerases IV and V, induction of the SOS response (to produce sufficient amounts of these polymerases), and induction of the RpoS response (described in detail later), which plays a crucial, but unidentified role. Using single-molecule fluorescence microscopy, the activation of pols IV and V in cells exposed to ciprofloxacin has now been observed directly (Figures 2E,F and section below; Henrikus et al., 2018). Fourth, homologous recombination is the primary mode of repair for double-strand breaks in *E. coli* (Dillingham and Kowalczykowski, 2008). The presence of multiple double-strand breaks in ciprofloxacin-treated cells (Figure 2D), together with the presence of multiple chromosome equivalents (Figure 2C), means that high levels of homologous recombination are likely to take place between chromosome equivalents. Inter-chromosomal homologous recombination is thought to be mediated through the formation of large structures by the RecA protein (Figures 2G,H; Levin-Zaidman et al., 2000; Renzette et al., 2005; Lesterlin et al., 2014; Ghodke et al., 2019). Inter-chromosomal recombination has the potential to remove mutations, to introduce mutations (through error-prone break repair), and to combine mutations that originate within different chromosome variants into a single chromosome (Guttman and Dykhuizen, 1994; Lopez et al., 2007; Lopez and Blazquez, 2009; Pribis et al., 2019). In *E. coli*, inter-chromosomal recombination is strongly stimulated by exposure to ciprofloxacin (Lopez et al., 2007; Lopez and Blazquez, 2009), but not by antibiotics outside of the fluoroquinolone class (Lopez and Blazquez, 2009).

While the phenomena described above provide valuable insight into events in cells that accompany antibiotic-induced mutagenesis, they are by no means the only factors involved. Environmental factors, including media composition, can dramatically impact mutagenesis (Maharjan and Ferenci, 2017). The intracellular production of ROS is demonstrated to be important for mutagenesis induced by ciprofloxacin (Pribis et al., 2019; Rodriguez-Rosado et al., 2019) and is highly likely to be involved in mutagenesis induced by other antibiotics (Kohanski et al., 2010; Thi et al., 2011). Interestingly, while ciprofloxacin-induced mutagenesis is largely ROS-dependent (Pribis et al., 2019; Rodriguez-Rosado et al., 2019), the mutation signatures produced do not match the expected signature for incorporation of 8-oxoguanine, an aberrant nucleotide that is highly mutagenic toward DNA replication *in vitro* and is known to accumulate in high ROS cells (Song et al., 2016). This suggests that the primary contribution of ROS production toward ciprofloxacin-induced mutagenesis is the triggering of mutagenic DNA repair pathways, as opposed to mutagenesis that results directly from oxidation of the nucleotide pool.

## Molecular Mechanisms of Antibiotic-Induced Mutagenesis: Activation of SOS and Error-Prone DNA Polymerases

The most widely studied form of antibiotic-induced mutagenesis is associated with the SOS response and involves increased production of error-prone DNA polymerases. *E. coli* produce three SOS-induced DNA polymerases (pols), pol II, pol IV, and pol V (Goodman and Woodgate, 2013). Of these, pols IV and V appear to be most responsible for SOS-dependent antibiotic-induced mutagenesis, although pol II can contribute to mutagenesis in some circumstances (Cirz et al., 2005; Pribis et al., 2019).

Pol V is the most error-prone DNA pol produced in *E. coli* cells (Jaszczur et al., 2016). Its activity is regulated at multiple levels, ensuring that its activity in the absence of exogenous DNA damage is minimal (Goodman et al., 2016). At least three steps in the pol V regulation pathway are dependent on RecA* nucleoprotein filaments (multimeric filaments of RecA on single-stranded DNA), which are produced during the repair of single-stranded DNA gaps and double-strand breaks *via* homologous recombination (Goodman et al., 2016). First, pol V is assembled from the products of an SOS-regulated operon, *umuDC* (Tang et al., 1999; Fernandez de Henestrosa et al., 2000). Induction of the SOS response occurs when RecA* nucleoprotein filaments induce autocatalytic cleavage of the SOS repressor protein LexA (Maslowska et al., 2019). Thus, the UmuD_2_ (UmuD homodimer) and UmuC proteins are only expressed once RecA* accumulates in cells. At this stage, however, UmuD_2_ and UmuC do not form an active polymerase. Second, UmuD_2_ homodimers undergo autocatalytic cleavage in the presence of RecA*, in a mechanism analogous to that of LexA (Goodman et al., 2016). This cleavage produces the shorter protein UmuD'_2_. Whereas UmuD_2_ and UmuD'_2_ are relatively stable, the intermediate heterodimer form UmuDD' is rapidly degraded by the ClpXP protease, which imparts a form of threshold on the RecA*-mediated conversion of UmuD_2_ to UmuD'_2_ (Gonzalez et al., 1998). Third, RecA* is again required to form the active version of pol V, known as pol V Mut. UmuD'_2_ and UmuC physically interact to form pol V; however, this complex is a very poor polymerase (note that in this context pol V specifically refers to the complex UmuD'_2_C; Goodman et al., 2016). In a remarkable feat of biochemistry, pol V next locates a RecA* filament and extracts a single RecA protomer, with ATP intact, from the 3' proximal end (Goodman et al., 2016). This process forms the mutagenically active complex pol V Mut (UmuD'_2_C-RecA-ATP; Jiang et al., 2009). Once formed, pol V Mut remains active for only a few seconds before cleaving the ATP (*via* intrinsic ATPase activity; an unprecedented function among polymerases; Erdem et al., 2014; Jaszczur et al., 2019), at which point the complex becomes inactive (Goodman et al., 2016).

**Figure 3:**
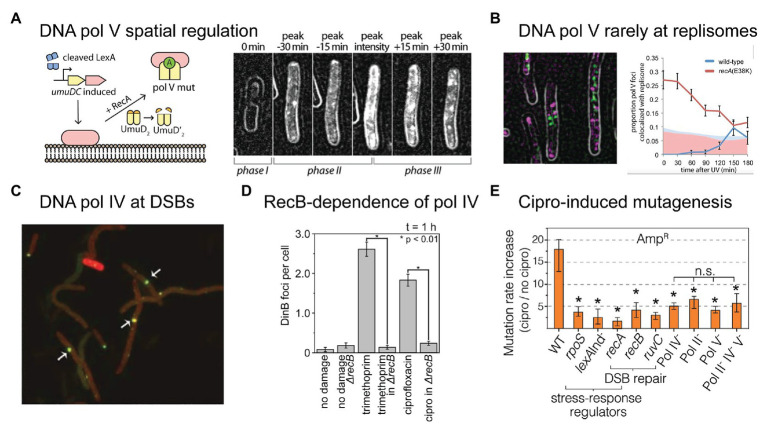
Recent insights into the activities of error-prone DNA polymerases in bacterial cells. **(A,B)** Single-molecule fluorescence microscopy analysis of DNA polymerase V in *E. coli* cells carrying UV-induced DNA damage. Figures adapted from Robinson et al. (2015). **(A)** DNA polymerase V is a rare example of a spatially regulated polymerase enzyme in bacteria, with intermediate forms being localized to the cell membrane and only the fully activated form being allowed to redistribute into the cytosol. The active form of DNA polymerase V, pol V Mut, is comprised of two molecules of UmuD', one molecule of UmuC, one molecule of RecA, and one molecule of ATP (i.e., UmuD'_2_-UmuC-RecA-ATP; Jiang et al., 2009). The formation of pol V Mut is regulated at several levels, in order to minimize pol V-dependent mutagenesis in the absence of DNA damage (Goodman et al., 2016). All stages of pol V regulation involve activities of RecA* nucleoprotein filaments. A single-molecule fluorescence microscopy analysis of cells expressing a fluorescent protein fusion of UmuC (UmuC-mKate2), revealed three stages in the activation of pol V in cells treated with a burst of UV light (Robinson et al., 2015). In phase I, cells began to grow into filaments, indicating induction of the SOS response, but produced little UmuC-mKate2. In phase II, cells began to express UmuC-mKate2. In this phase, UmuC-mKate2 was localized to the cell membrane. Due to the small number of UmuC-mKate2 features present in each cell, this can be difficult to see in individual representative images, but becomes abundantly clear in statistical analyses of cells across multiple images. Membrane localization is most evident in the image labeled “peak intensity”, where clear membrane-associated signals are visible along the edge of the cell. Finally, in phase III, UmuC-mKate2 was released into the cytosol as pol V Mut, where it formed punctate foci on the nucleoid, indicative of DNA synthesis occurring. This can be seen most clearly in the image labeled “peak + 30 min”. Release of UmuC from the membrane requires the cleavage of the UmuD protein to its shorter form UmuD'. **(B)** Pol V Mut rarely colocalises with replisomes. Contrary to existing models of pol V-dependent DNA synthesis, in which pol V carries out translesion synthesis at stalled replication forks, single-molecule fluorescence imaging revealed that pol V Mut rarely colocalises with replication forks. This indicates that pol V likely works on other DNA substrates in cells, which may include single-stranded gaps and/or recombination intermediates. **(C–E)** DNA polymerase IV (pol IV) works on double-strand break repair intermediates in cells treated with ciprofloxacin. **(C)** Single-molecule fluorescence microscopy demonstrated that fluorescently labeled pol IV forms foci that colocalise with sites of double-strand breaks induced by the SceI restriction enzyme. Figure adapted from Mallik et al. (2015). **(D)** A second single-molecule imaging study demonstrated that the formation of pol IV foci in cells treated with either ciprofloxacin or trimethoprim requires processing of double-strand breaks by the RecBCD complex. Figure adapted from Henrikus et al. (2020). This, together with other evidence, indicates that these foci likely represent pol IV working on recombination intermediates during double-strand break repair. **(E)** Ciprofloxacin-induced mutagenesis is a form of error-prone double-strand break repair, requiring induction of the SOS and RpoS responses (regulated by *lexA* and *rpoS*), double-strand break repair (dependent on *recA*, *recB*, *recC*, and *ruvC*), and error-prone DNA polymerases (Pols II, IV, and V). Figure adapted from Pribis et al. (2019). Crucially, the RpoS response is only induced in a sub-population of cells, indicating that ciprofloxacin-induced mutation rates are elevated within only a fraction of the cell population (~10%).

Microscopy is already beginning to play a major role in improving our understanding of these polymerases in bacteria (Joseph and Badrinarayanan, 2020). Using single-molecule fluorescence microscopy, we visualized the RecA*-dependent activation of pol V in cells treated with DNA damaging agents, including ciprofloxacin (Robinson et al., 2015; Henrikus et al., 2020). In doing so, we discovered yet another element that controls pol V activity – spatial regulation (Figure 3A; Robinson et al., 2015). While spatial regulation has been well-established in eukaryotic systems, this was the first example of a DNA-acting enzyme being spatially regulated in bacteria. In the case of pol V, spatial regulation is imposed *via* an interaction between UmuC and the inner cell membrane. Following SOS-induced expression of UmuD_2_ and UmuC, but prior to UmuD′_2_ being formed, UmuC associates with the membrane. Once UmuD′_2_ is formed, UmuC is released from the membrane as part of either pol V (UmuD′_2_C) or pol V Mut (UmuD′_2_C-RecA-ATP). It remains unknown whether pol V Mut formation occurs on the membrane or in the cytosol. In the microscopy images, pol V spatial regulation presents as a redistribution of fluorescently labeled UmuC from the membrane to the cytosol. Due to the fact that UmuC is expressed at very low levels in *E. coli* (0–15 mol/cell; Robinson et al., 2015), UmuC produced patchy signals and this spatial redistribution was difficult to observe by eye, but was well-supported by statistical analyses. In subsequent work, we developed a novel image-analysis technique that enabled us to extract the relative proportion of membrane-bound and cytosolic UmuC as a function of time (Goudsmits et al., 2016). As UmuC levels peak, 90% of the protein is associated with the membrane. Within 30 min, this proportion decreases to 50%, with the remaining 50% now appearing in the cytosol. As pol V Mut is formed and begins to work on the DNA within the cytosol, punctate foci of UmuC are formed. This phenomenon is known as detection by localization and occurs because the rate of diffusional motion slows dramatically once the protein binds to the DNA. Interestingly, we observed that pol V Mut foci formed at sites on the nucleoid that were spatially distinct from replisomes (Figure 3B). This strongly suggests that pol V Mut acts on non-replisomal substrates, such as single-stranded DNA gaps or homologous recombination intermediates (Robinson et al., 2015). In a follow-up study, we observed that the number of pol V Mut foci formed in ciprofloxacin-treated cells did not appear to correlate with the number of double-strand breaks present, indicating that the substrates for pol V Mut are unlikely to be homologous recombination intermediates formed during double-strand break repair (Henrikus et al., 2020).

In contrast, recent studies have revealed a clear role in double-strand break repair for the other error-prone polymerase present in *E. coli*, pol IV. A role for pol IV in (error-prone) break repair was originally discovered through genetics experiments (Ponder et al., 2005). The single-molecule imaging measurements described below demonstrated that break-repair intermediates are the major substrates for pol IV in cells treated with (fluoro)quinolone antibiotics.

While many had tried, the Foster group was the first to produce a functional fluorescent protein fusion of pol IV (Mallik et al., 2015). This was a considerable achievement as assaying for pol IV-dependent activities in cells is non-trivial. Furthermore, the activity of fusions turns out to be highly sensitive to the length of the linker used to connect the pol IV protein (DinB) to the fluorescent protein. Using the active (20 amino-acid linker) fusion they showed, among other things, that pol IV localizes as punctate foci in cells treated with the quinolone antibiotic naladixic acid (Mallik et al., 2015). These foci tightly colocalised with RecA* foci, which are expected to form at sites of double-strand breaks. Using a restriction enzyme to induce a single break within the chromosome, they observed that pol IV localized to the break site (Figure 3C). Building on this work, we showed that the binding of pol IV to the nucleoid in cells treated with ciprofloxacin or trimethoprim was strongly dependent on a gene (*recB*) involved in double-strand break repair (Henrikus et al., 2020). Disruption of the RecBCD complex, which carries out the early stages of double-strand break repair, almost completely abolished the formation of pol IV foci in cells treated with either ciprofloxacin or trimethoprim (Figure 3D). Together with existing evidence from genetics and biochemistry studies (Ponder et al., 2005; Williams et al., 2010; Shee et al., 2011a,b, 2012; Pomerantz et al., 2013a,b; Tashjian et al., 2019), these microscopy observations indicated that a major role of pol IV in cells is to act in double-strand break repair. The likely function for pol IV in this context is the extension of D-loop structures.

Using a combination of microscopy and classical genetics approaches, the Rosenberg group recently demonstrated that ciprofloxacin-induced mutagenesis is ROS-dependent, and is driven by error-prone double-strand break repair (Pribis et al., 2019). Ciprofloxacin-induced mutagenesis was found to be dependent on genes encoding pols II, IV, and V (*polB*, *dinB*, and *umuDC*), double-strand break repair factors (*recA*, *recB*, and *ruvC*), the SOS response (mutagenesis was blocked in a *lexA* [Ind^−^] background), and the RpoS response (Figure 3E; the RpoS response is introduced in greater detail in the next section). The authors discovered that ROS levels were elevated in a sub-population (~10%) of ciprofloxacin-treated cells. Elevated mutagenesis was strongly enriched within this sub-population. In this context, the main function of ROS is to induce the RpoS response; mild overexpression of RpoS alleviated the requirement for ROS in the mutagenesis assays. The observation of an RpoS-high sub-population is an example of phenotypic heterogeneity. Microscopy is revealing phenotypic heterogeneity to be a common theme bacterial DNA repair and mutagenesis (Vincent and Uphoff, 2020). Improving our understanding of population heterogeneities is vital if we are to better understand antibiotic-induced mutagenesis.

Whereas, the Rosenberg study points to pol IV-dependent error-prone break repair occurring in a subset of high-ROS, RpoS-positive cells (Pribis et al., 2019), our imaging study showed that ciprofloxacin-induced pol IV foci formed in all cells (Henrikus et al., 2020). The model that is most consistent with the data is that pol IV is involved in double-strand break repair in all cells and that this somehow becomes error-prone in the RpoS-positive sub-population. In other words, the RpoS response modulates the fidelity of pol IV, rather than its ability to engage double-strand break repair intermediates. The role of RpoS in mutagenesis remains unknown, however, it is tempting to speculate that RpoS-dependent modulation of mismatch repair could play a role, as has been reported in other systems (Gutierrez et al., 2013).

## Molecular Mechanisms of Antibiotic-Induced Mutagenesis: RpoS Response and Regulation of Mismatch Repair

Using classical genetics approaches, the Matic group discovered that cells treated with the β-lactam antibiotic ampicillin induced pol IV-dependent mutagenesis (Gutierrez et al., 2013). As with the ciprofloxacin-induced mutagenesis (Pribis et al., 2019) described in the previous section, the RpoS response was found to play a key role in ampicillin-induced mutagenesis (Gutierrez et al., 2013). The RpoS response (also known as the σ^S^ response or general stress response) is induced under a variety of circumstances, including stationary growth, starvation, and oxidative damage (Battesti et al., 2011; Gottesman, 2019). The protein RpoS (or σ^S^) is an alternative σ-factor that governs the expression of hundreds of different genes (Battesti et al., 2011). When expressed, RpoS competes with the housekeeping σ-factor, σ^70^ (or RpoD), for binding to RNA polymerase, and in doing so dramatically changes the gene expression profile of the cell (Battesti et al., 2011).

The Matic group showed that representative quinolone, β-lactam, and aminoglycoside antibiotics induced the RpoS response (Gutierrez et al., 2013). Choosing to investigate ampicillin in greater depth, they demonstrated that ampicillin-induced mutagenesis required both pol IV and induction of the RpoS response. Unlike ciprofloxacin-induced mutagenesis, induction of the SOS response was not required for ampicillin-induced mutagenesis. They also observed that mild overexpression of the mismatch repair protein MutS (which functions to recognize nucleotide mismatches) completely suppressed mutagenesis. This led to the discovery of a small regulatory RNA, SdsR, which is expressed during the RpoS response and reduces the expression of MutS. They proposed a model in which depletion of mismatch repair leads to less efficient repair of mismatches produced by pol IV, which in turn leads to increased mutagenesis.

Representative quinolones and aminoglycosides also induce the RpoS response (Gutierrez et al., 2013) and exhibit pol IV-dependent mutagenesis (Thi et al., 2011). It is reasonable to speculate that the depletion-of-mismatch-repair mechanism at play during ampicillin-induced mutagenesis may extend to other bactericidal antibiotics. A single-molecule/single-cell imaging approach that would seemingly provide the ideal platform on which to test this idea has already been developed.

Elez developed an approach that allowed mismatches to be visualized by microscopy (Elez et al., 2012). By fluorescently labeling the mismatch repair protein MutL (the molecular coordinator of mismatch repair), it became possible to visualize the process of mismatch detection by the MutS and MutL proteins. Each time a mismatch was detected, a punctate MutL focus became visible in microscope images. In a series of later studies, Elez and colleagues repeated the measurements in cells that lacked the *mutH* gene, which is required for repair of the mismatch (Elez et al., 2010, 2018; Robert et al., 2018). This ensured that the mismatch would be fixed to a mutation in the next round of DNA replication, providing a means to infer mutation rates in real-time.

**Figure 4:**
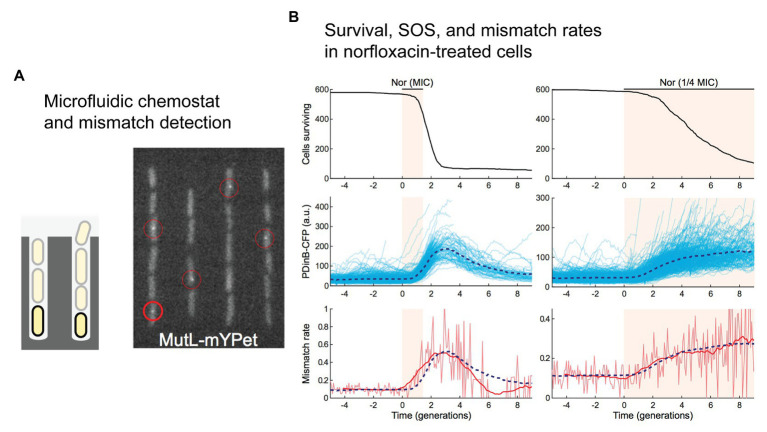
Direct detection of mismatches in *E. coli* cells treated with norfloxacin. **(A,B)** A single-molecule fluorescence microscopy study of cells growing in microfluidic chemostats revealed an increased rate of mismatches in cells treated with the fluoroquinolone antibiotic norfloxacin. Figures adapted from Uphoff (2018). **(A)** A microfluidic “mother machine” was used to monitor individual cells through multiple generations. A fluorescent protein fusion of the mismatch repair protein MutL (MutL-mYPet) allowed mismatches to be visualized as punctate foci within cells. **(B)** Treatment of cells with norfloxacin induces a substantial increase in mismatch rate. Left unrepaired, these mismatches would become mutations during the subsequent round of DNA replication. The dynamics of mismatch formation match well with induction of the SOS response, which is monitored in this study by expression of cyan fluorescent protein (CFP) from the SOS-inducible *dinB* promoter (pDinB-CFP). Importantly, both SOS induction and the elevated mismatch rate lag behind cell death – increased mutagenesis is likely to occur in a small number of cells that survive beyond the initial stages of treatment.

While it was not the primary goal his study (Uphoff, 2018), Stephan Uphoff used the labeled-MutL system to study mismatch-detection rates in cells treated with the fluoroquinolone antibiotic norfloxacin (Figure 4). Growing the cells in a single-cell chemostat device called a “mother machine” (Figure 4A; Wang et al., 2010), he observed a significant increase in the number of mismatches detected in cells by fluorescent MutL (Uphoff, 2018). Delivering a pulse of norfloxacin at a concentration equal to the MIC, a transient 5-fold increase in the number of detected mismatches was observed (Figure 4B). Delivering a lower concentration (0.25x MIC) for a longer time led to a more sustained elevation (3-fold) of the mismatch-detection rate. In both experiments, induction of the SOS was also measured, revealing that changes in the mismatch-detection rate correlated with induction of the SOS response. The MutL-based mismatch-detection system could be an extremely useful tool in the study of antibiotic-induced mutagenesis. In particular, it would allow the role of RpoS-based regulation of mismatch repair to be assessed much more broadly and at an unprecedented level of detail. Each MutL focus represents a mismatch that would normally be repaired before becoming fixed as a mutation. One would hypothesize that disruption of the RpoS response would prevent the down-regulation of MutS, leading to more efficient detection of mismatches and a corresponding increase in the number of MutL foci detected in cells treated with antibiotics.

## Measurements Linking Mutations to Cell and Population Outcomes

**Figure 5:**
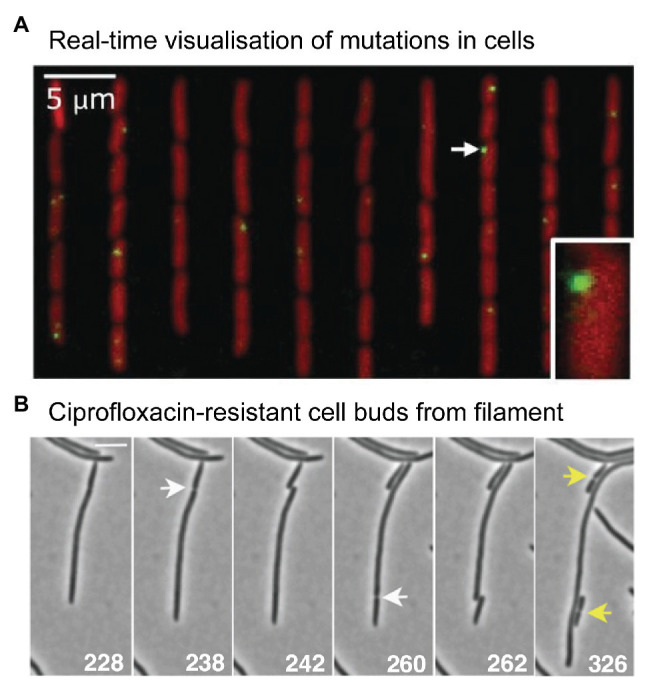
Microscopy measurements linking mutations with phenotypic changes. **(A)** Direct measurement of a distribution of fitness effects from *E. coli* cells growing in a microfluidic chemostat. Figure adapted from Robert et al. (2018). Mismatches were detected using a fluorescent protein fusion of the mismatch repair protein MutL (YFP-MutL). Foci were produced in cells whenever YFP-MutL bound to a mismatch. The cells also carried a deletion of the *mutH* mismatch repair gene, ensuring that all mismatches became mutations in the subsequent round of DNA replication. The experimenters were able to link the appearance of mutations with changes in cell growth rates. They were able to produce a well-sampled distribution of fitness effects. Contrary to the dogma that most mutations are deleterious, the distribution showed that the vast mutations were quasi-neutral, having very little effect on growth. Only 1% of mutations were lethal. **(B)** Time-lapse microscopy captures the moment a ciprofloxacin-resistant cell buds from filamentous, multinucleate *E. coli* cell growing in a sub-inhibitory concentration of ciprofloxacin. Figure adapted from Bos et al. (2015).

The mismatch-detection approach developed by Elez would also be an ideal platform to begin connecting the molecular world of mutagenesis with changes at the cell and population levels. In fact, her group has already taken steps in this direction, measuring a key population-level descriptor, the distribution of fitness effects, and for cells growing in microfluidic mother machines (Robert et al., 2018). These devices allow for long-term measurements to be made under precisely controlled experimental conditions. The group monitored mismatch detection by MutL in a mismatch repair-defective *mutH* strain so that mismatches would be fixed as mutations in subsequent round of DNA replication (Figure 5A). In parallel, they tracked cell growth rates, enabling them to link the appearance of spontaneous mutations in cells with changes in growth rate. Because the cells were physically isolated from each other within the mother machine, there was no competition between variants in the population and thus, growth rate measurements directly reflected the fitness of the mutants in the conditions tested. Approximately, ~20,000 individual mutation events were detected over 200 generations. The measurements revealed that the majority of mutations observed in this experiment were quasi-neutral, having little, or no effect on fitness. Only 1% of mutations were lethal.

Accurately measured distributions of fitness effects are key to evolutionary modeling and would be particularly advantageous in the modeling of antibiotic-resistance development. If the mother machine format could be adapted for use with antibiotics, this might even represent a route toward a quantitative assessment of the role of antibiotic-induced mutagenesis in the evolution of antibiotic resistance. Because the technique is based on microscopy, multiple facets of antibiotic-induced mutagenesis could be monitored in parallel. In addition to mismatch rates and cell growth rates, cell filamentation and cell death could also be monitored. With appropriate probes already in hand, it should be also possible to observe induction of the SOS and RpoS responses and corresponding activities of error-prone pols IV and V.

What might evolution to antibiotic resistance look like under the microscope? A 2015 study by Bos et al. (2015) provides some clues. As highlighted earlier (Figure 2), the authors observed that cells treated with ciprofloxacin at 0.125x MIC grow into filaments that contain multiple nucleoid masses (Bos et al., 2015). Occasionally, the authors observed regular-sized cells budding from the tip of these filaments (Figure 5B). Most of the time these buds were inviable, however, buds were occasionally produced that grew normally. These cells did not grow into filaments, indicating that they had become resistant to the antibiotic. Expansion of these resistant variants at 0.5x MIC or 1x MIC ciprofloxacin, followed by sequencing of the *gyrA* and *marR* loci, confirmed the presence of resistance mutations in many of the variants (in other cases, resistance was assumed to occur *via* mutations at other loci that were not sequenced). Expansion at 1x MIC strongly favored the *gyrA* (S83A) mutation, which appeared in 7/8 isolates. The Bos et al. (2015) study indicates that antibiotic-resistant variants can appear frequently enough to be detected *via* microscopy.

## Measurements Linking Cell Growth Dynamics to Population Outcomes

**Figure 6:**
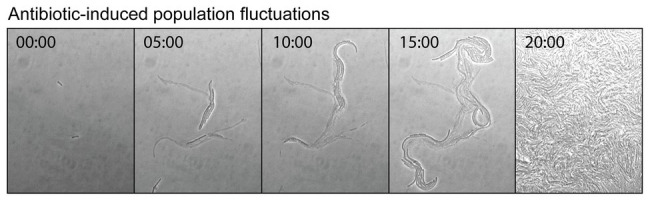
Antibiotic-induced population fluctuations. It is well known that when bacterial growth is monitored as a function of antibiotic concentration, population growth rates tend to slow as the minimum inhibitory concentration (MIC) is approached. Recently, it was discovered that for bactericidal antibiotics this reduction in the net growth of the population is caused not by the slowing of cell division, but by some of the cells dying (Coates et al., 2018). As the MIC is approached, the rate of cell death approaches that of cell division. This is neatly visualized by a time-lapse movie, which shows cells undergoing stochastic cell death when growing at a concentration of cefsulodin equivalent to 0.8x MIC. Live and dead cells can be distinguished by contrast. The live cells produce high contrast in the bright-field images, whereas the dead cells, which have lysed and have left behind an empty cell-shaped sac, produce only low image contrast. At various points in the movie, a portion of the population undergoes stochastic cell death. These population fluctuations have far-reaching implications for the study of antibiotic-induced mutagenesis and for its potential to supply antibiotic resistance mutations. Figure adapted from Coates et al. (2018).

Antibiotics can induce mutagenesis, tolerance, and cell death. Evolution to antibiotic resistance is often limited by the mutation supply rate, which is a product of the cellular mutation rate and the size of the population (Hughes and Andersson, 2017). Elevated mutagenesis acts to increase the mutation supply, while cell death reduces it by decreasing the size of the population. Seeking to minimize the effects of cell death, antibiotic-induced mutagenesis is typically studied using sub-MIC concentrations of antibiotic. Importantly, it was demonstrated recently that significant cell death still occurs in this sub-MIC regime. Through a combination of microscopy, plate-reader assays, and modeling, the Kim group investigated the extinction of small populations of cells (<100) upon treatment with antibiotics (Coates et al., 2018). Their analysis revealed that bactericidal antibiotics induced stochastic fluctuations in population size. This was visualized by time-lapse microscopy images of cells growing in the presence of the cephalosporin antibiotic cefsulodin, at a concentration equivalent to 0.8x MIC (Figure 6). During the course of the time-lapse measurement, the total number of cells that are present increases, however, this occurs more slowly than in the absence of drug because many of the cells die. Stochastic cell death leads to marked fluctuations in the population size as a function of time. This simple observation has broad ramifications for the study of antibiotic-induced mutagenesis.

The fact that population growth in this near-MIC regime is the product of both cell growth and cell death means that many more generations are required for the population to reach a certain size than would be required in the absence of drug. When rates of antibiotic-induced mutagenesis are measured using fluctuation analyses, the results are reported in units of mutations per genome per generation. Typically, the number of generations is calculated based on the population size after treatment, under the assumption that cells are not dying when the antibiotic concentration is below the MIC. In light of the stochastic population dynamics reported by the Kim group, it appears that many of the mutation rates reported in the literature may be overestimated because more generations occurred than were accounted for in the measurements (Frenoy and Bonhoeffer, 2018).

The second important ramification of stochastic population dynamics for the study of antibiotic-induced mutagenesis is that when the population size is small, as would be the case in a mother machine for example; many populations will become extinct when exposed to sub-MIC concentrations of bactericidal antibiotics. Evidence of this is visible in Uphoff’s work with sub-MIC norfloxacin (Uphoff, 2018), where the number of viable cells decreases continuously throughout the experiment (Figure 4B). Presumably, this has occurred because of the small and finite size of the channels in the mother machine. Cells that are growing do not increase the size of the population being monitored because cells that no longer fit in the channel are washed away and thus are no longer monitored. Cells that die decrease the size of the observed population. Eventually, none of the channels would contain any viable cells. All of this occurred more quickly in cells treated with norfloxacin concentrations equivalent to the MIC because cells die more often. Because Uphoff monitored mismatch formation and cell death simultaneously we can carry out a putative assessment of the effects of norfloxacin exposure on mutation supply. In measurements with 1x MIC norfloxacin, mismatch-detection rates increased while cell viability fell (Figure 4B). At its peak, mismatch-detection rates were ~5x above basal levels (Figure 4B; lower left panel; three generation times). At this time point, however, survival had dropped to 10% of the original value (Figure 4B; upper left panel; three generation times). If we take the product of these two values as a crude approximation of the mutation supply rate within the mother machine device, mutation supply would have fallen by 50% in the norfloxacin-exposed population. In the measurements with 0.25x MIC norfloxacin, cell viability decreased more slowly (Figure 4B; upper right panel). By the five generations time-point, viability had reduced to 50% (Figure 4B; upper right panel), while the mismatch-detection rate had increased ~2.5x (Figure 4B; lower right panel). Thus, the mutation supply at this time would be predicted to be higher (125%) than that of the initial population. At a later time-point (eight generation times), the mutation supply would have been lower than the starting population; the mismatch-detection frequency had increased 3x, however, cell survival was only ~15%. The effects of cell death are amplified in the mother machine format, however, the fact that mismatch-detection frequencies and cell survival rates change as a function of time after drug exposure highlight the need for simultaneous, real-time measurements of mutation rates, and cell growth/death parameters. Such measurements are vital if we are to improve our understanding of antibiotic-induced mutagenesis. Bacterial population sizes are also affected by non-genetic phenomena, such as antibiotic tolerance and persistence. Accurate measurements of cell growth and survival would also provide new opportunities to assess the roles of antibiotic tolerance and persistence in the development of antibiotic resistance mutations.

## Concluding Remarks

The studies highlighted throughout this review demonstrate the potential of microscopy in helping to achieve a more thorough understanding of antibiotic-induced mutagenesis. There is enormous potential for other types of correlative measurements as well. Microscopy shows a great degree of promise as an important platform for uncovering functional linkages between the molecular-level actions of error-prone DNA repair processes, the cell-level behaviors of stress responses and growth dynamics, and population-level behaviors that determine mutation supply rates. The development of advanced techniques for microscopy and microfluidic technology is sure to continue, however, we feel it is important to point out that great strides can be taken with the technology that is already available. As has been noted previously (Potvin-Trottier et al., 2018), our understanding of other antibiotic-induced phenomena, including tolerance and persistence, would also strongly benefit from further application of state-of-the-art microscopy and microfluidic technologies.

## Author Contributions

All authors listed have made a substantial, direct and intellectual contribution to the work, and approved it for publication.

### Conflict of Interest

The authors declare that the research was conducted in the absence of any commercial or financial relationships that could be construed as a potential conflict of interest.
